# Integrins Cooperate With the EGFR/Ras Pathway to Preserve Epithelia Survival and Architecture in Development and Oncogenesis

**DOI:** 10.3389/fcell.2022.892691

**Published:** 2022-06-13

**Authors:** Andrea Valencia-Expósito, M. Jesús Gómez-Lamarca, Thomas J. Widmann, María D. Martín-Bermudo

**Affiliations:** ^1^ Centro Andaluz de Biología del Desarrollo CSIC-Universidad Pablo de Olavide, Sevilla, Spain; ^2^ Departamento de Biología Celular, Universidad de Sevilla, Sevilla, Spain; ^3^ GENYO, Granada, Spain

**Keywords:** integrins, survival, JNK, EGFR, oncogenesis

## Abstract

Adhesion to the extracellular matrix (ECM) is required for normal epithelial cell survival. Disruption of this interaction leads to a specific type of apoptosis known as anoikis. Yet, there are physiological and pathological situations in which cells not connected to the ECM are protected from anoikis, such as during cell migration or metastasis. The main receptors transmitting signals from the ECM are members of the integrin family. However, although integrin-mediated cell-ECM anchorage has been long recognized as crucial for epithelial cell survival, the *in vivo* significance of this interaction remains to be weighed. In this work, we have used the *Drosophila* wing imaginal disc epithelium to analyze the importance of integrins as survival factors during epithelia morphogenesis. We show that reducing integrin expression in the wing disc induces caspase-dependent cell death and basal extrusion of the dead cells. In this case, anoikis is mediated by the activation of the JNK pathway, which in turn triggers expression of the proapoptotic protein Hid. In addition, our results strongly suggest that, during wing disc morphogenesis, the EGFR pathway protects cells undergoing cell shape changes upon ECM detachment from anoikis. Furthermore, we show that oncogenic activation of the EGFR/Ras pathway in integrin mutant cells rescues them from apoptosis while promoting their extrusion from the epithelium. Altogether, our results support the idea that integrins promote cell survival during normal tissue morphogenesis and prevent the extrusion of transformed cells.

## Introduction

To survive or die is a cellular decision controlled by environmental cues and physical stimuli. This life and death decision is deeply influenced by the extracellular matrix (ECM) (Meredith et al., 1993). When cells lose their normal interactions with the ECM, the cell cycle is arrested and a specific form of apoptosis, known as anoikis, is initiated (Frisch and Ruoslahti, 1997). During development, aberrant anoikis alters tissue architecture and function, compromising embryonic viability (Mole et al., 2021). Conversely, programmed anoikis contributes to physiological developmental processes and tissue renewal (Gilmore, 2005; Chiarugi and Giannoni, 2008). Anoikis is also an important surveillance mechanism, ensuring that any cell that loses its appropriate position within a tissue is targeted for death. For this reason, impaired anoikis contributes to the malignancy of many cancer cells, allowing them to survive without ECM anchorage and facilitating their dispersion [reviewed in (Zhong and Rescorla, 2012)]. Unraveling the mechanisms regulating anoikis is therefore crucial to understand both normal morphogenesis and cancer progression.

Although the concept of anchorage-dependent cell survival has been recognized for many years, it was in the early 1990’s when it was demonstrated that cells deprived of ECM attachment undergo anoikis (Meredith et al., 1993; Frisch and Francis, 1994). Anoikis can be considered a multistep process [reviewed in (Zhong and Rescorla, 2012)]. First of all, loss of anchorage results in inactivation of focal complex signalling molecules, including FAK and Src (Giancotti, 2000; Parsons, 2003). As a consequence, several pro-survival pathways such as PI3K/Akt and Raf/MEK/ERK are disrupted [reviewed in (Vachon, 2011)]. The second step in anoikis is the simultaneous disassembly of focal adhesions and the destabilization of the cytoskeleton, which leads to the release of pro-apoptotic Bcl-2 proteins (Martin and Vuori, 2004). The final step is the activation of apoptotic kinases, including JNK, which results in an increase in the expression and phosphorylation of the pro-apoptotic BH3-only proteins BIM an BMF (Girnius and Davis, 2017). However, the role of JNK in anoikis remains controversial, as, depending on the cell type, kinase isoform and stimulus, it can perform pro- or anti-apoptotic functions (Liu and Lin, 2005). Analysing JNK function in different cellular contexts shall help resolving its debated role in anoikis.

The major transmitters of survival signals from the ECM are members of the integrin family (Frisch and Ruoslahti, 1997; Meredith et al., 1993; Vachon, 2011). Integrins are a widely expressed family of heterodimeric transmembrane glycoproteins, composed of an *a* and a β subunit, that link the ECM to the actin cytoskeleton (Hynes, 1992). The vertebrate integrin family is comprised of 18 *a* and 8 β transmembrane subunits enabling about 24 different heterodimeric receptors for a diverse array of ECM proteins. However, only some integrins are capable of regulating cell viability and these include the β1 integrin subfamily (Stupack and Cheresh, 2002). The regulation of cell survival mediated by integrins implicates an increasing complexity of players depending on the tissue, cell type, specie and the cell differentiation state, emphasizing the need to study anoikis in a given tissue within its physiological context [reviewed in (Ignotz and Massague, 1987; Frisch and Screaton, 2001; Vachon, 2011)]. Nonetheless, the early embryonic lethality observed in some vertebrate models lacking β1 integrins has long precluded a direct evidence for a role of β1 integrins as survival factors *in vivo* (Stephens et al., 1995). The use of cell lineage-specific gene deletion approaches and transplantation experiments to circumvent early embryonic lethality has revealed that the role of integrin function in cell survival *in vivo* is multifaceted and sometimes contradictory. Thus, while depletion of β1 integrins from either the developing mouse lens, the epidermis or the endothelia results in apoptosis (DiPersio et al., 2000; Simirskii et al., 2007; Carlson et al., 2008), conditional deletion in the intestinal epithelium causes anoikis resistance, increased cell proliferation and defective differentiation (Jones et al., 2006). Furthermore, in some situations, cells are protected from anoikis, such as during cell migration or metastasis. In these cases, cells escape death using a variety of strategies, including hyperactivation of receptor tyrosine kinases (Guadamillas et al., 2011; Paoli et al., 2013).

Since the main components of the cell death machinery are evolutionary conserved*, Drosophila*, due to its unique genetic and cell biological advantages, has become an ideal model system to study apoptosis in the context of a developing organism. As in mammals, the caspase family of cysteine proteases is essential for the regulation of apoptosis also in *Drosophila*. These proteins are constitutively expressed as procaspases that have low but significant protease activity. To prevent unwanted consequences of basal procaspase activity, living cells express high levels of the Inhibitor of Apoptosis (IAP) proteins, Diap1 and Diap2, which act as E3-ubiquitin ligases and target activator and effector caspases for proteasome degradation [reviewed in (Hay, 2000)]. Upon an apoptotic stimulus, Diap1 antagonists, including *hid*, *reaper* (*rpr*), *grim*, *sickle* (*skl*), *jafrac2*, and *dOmi/HtrA2* are transcriptionally activated and physically interact with Diap1 [reviewed in (Xu et al., 2009)]. This leads to the blockage of its caspase inhibitory function and the degradation of Diap1 by auto-inhibitory ubiquitinization. As a consequence, the caspase cascade is activated and the apoptosome is formed. First of all, initiator pro-caspases, including DRONC, the casp9 ortholog, DREDD and DCP-2, are activated by auto-cleavage. Then, initiator caspases catalyse the activation of the effector pro-caspases drICE and DCP-1, and this in turn cleave their substrates, including cytoskeleton proteins and DNA metabolic proteins (Cooper et al., 2009). However, despite the conservation of the cell death response between flies and vertebrates, little is known about the process of anoikis in *Drosophila*.

Using the primordium of the *Drosophila* wing, the wing imaginal disc, as model system, we have studied the implication of integrins in the regulation of epithelial cell survival in the context of a developing tissue. The formation of the wing starts during early embryonic development when about 30 cells are allocated to form the wing imaginal disc primordium. During larval life, disc cells divide and form an epithelial sac comprised of a folded columnar epithelium on one side and a squamous epithelium on the other (Garcia-Bellido and Merrian, 1971). The mature disc will give rise to the adult wing and notum. At the onset of metamorphosis, local cell shape changes promote the folding of the single-layered columnar epithelium into a bi-layered sheet in which epithelial cells face each other along their basal sides (Fristrom et al., 1993). Integrins perform two distinct functions in the wing disc, to hold the two layers together and to regulate the cell shape changes underlying folding (Brabant et al., 1996; Dominguez-Gimenez et al., 2007). However, despite the known role of integrins as survival factors in both benign and malignant epithelia [reviewed in (Gilcrease, 2007)], integrins have not yet been implicated in cell survival in wing disc epithelial cells.

Here, we show that low integrin levels induces caspase-dependent wing disc cell death. This anoikis process is mediated by the activation of the JNK pathway, which in turn induces expression of the proapoptotic gene *hid*. We also find that integrins cooperate with the EGFR pathway to maintain wing disc epithelial cell survival. Finally, our results demonstrate that an oncogenic version of Ras, Ras^V12^, blocks anoikis in the wing disc and that elimination of integrins from transformed Ras^V12^ cells stimulates their basal extrusion from the wing disc epithelium. Our results unravel new roles for integrins in epithelial cells *in vivo*, promoting cell survival during tissue morphogenesis and preventing the extrusion of transformed cells.

## Materials and Methods

### Fly Strains

The following stocks were used: *UAS-Ras* ^*V12*^ (Lee et al., 1996); *nub*-Gal4 (Calleja et al., 1996); *hid* 5′F-GFP (Tanaka-Matakatsu et al., 2009); UAS-DER^DN^, *UAS-Diap1*, *UAS-puc*, *ap-Gal4*, *puc-LacZ*, *rpr-LacZ*, and *brk-LacZ* (Bloomington *Drosophila* Stock Center); *mys* ^*RNAi*^, UAS-diβ (Martin-Bermudo and Brown, 1999) and *hid* ^*RNAi*^ (Vienna *Drosophila* RNA-i Center). To generate mutant clones in the wing disc we used the FRT/FLP technique (Chou and Perrimon, 1992). Mutant clones were marked by the absence of GFP. The following mutant alleles and chromosomes were used: *mys* ^*11*^ [also known as *mys* ^*XG43*^ (Bunch et al., 1992)] and *e22c*-Gal4 UAS-*flipase* (Duffy et al., 1998). The *e22c*-Gal4 driver is expressed in some posterior wing disc cells and was therefore used in combination with UAS- *flp* to generate large *mys* clones. Flies were raised at 25°C.

### Immunohistochemistry and Imaging

Wing imaginal discs were stained using standard procedures and mounted in Vectashield (Vector Laboratories, Burlingame, CA, United States). The following primary antibodies were used: goat anti-GFP^FICT^ (Abcam, 1:500), rabbit anti-caspase Dcp1 (Cell Signaling; 1:100), rabbit anti-pJNK (Promega, 1:200), mouse anti-βGal (Promega, 1:1000), mouse anti-βPS (Developmental Studies Hybridoma Bank, DSHB, University of Iowa, United States, 1:50) and mouse anti-myc (Promega, 1:100). The secondary antibodies used were Alexa fluor 488, (Molecular Probes^TM^) and Cy3 and Cy5 (Jackson ImmunoReseach Laboratories, Inc.) at 1: 200. DNA was labelled using Hoechst (Molecular Probes, 1:1000). Confocal images were obtained using a Leica SP5-MP-AOBS, equipped with a Plan-Apochromat 40X oil objective (NA 1.4).

### Quantification of Fluorescence Intensity

To quantify fluorescent intensity of the different markers, fluorescent signaling was measured from maximum projections of 30 Z-confocal sections taken with 1 μm of interval per wing disc and genotype. Quantifications were made in the wing pouch region, which was manually selected using the FIJI-Image J line selection tool. Microscope settings were maintained between imaging sessions in each experiment. The background value taken from cell-free region was subtracted from all data series. Measurements of whole fluorescence intensity were done dividing the mean of all included pixels intensity by the outlined cell area, using the FIJI-Image J measure tool. Student’s t tests were used for statistical comparisons of fluorescence intensity values.

## Results

### Integrins are Required for the Survival of Wing Disc Epithelial Cells

**FIGURE 1:**
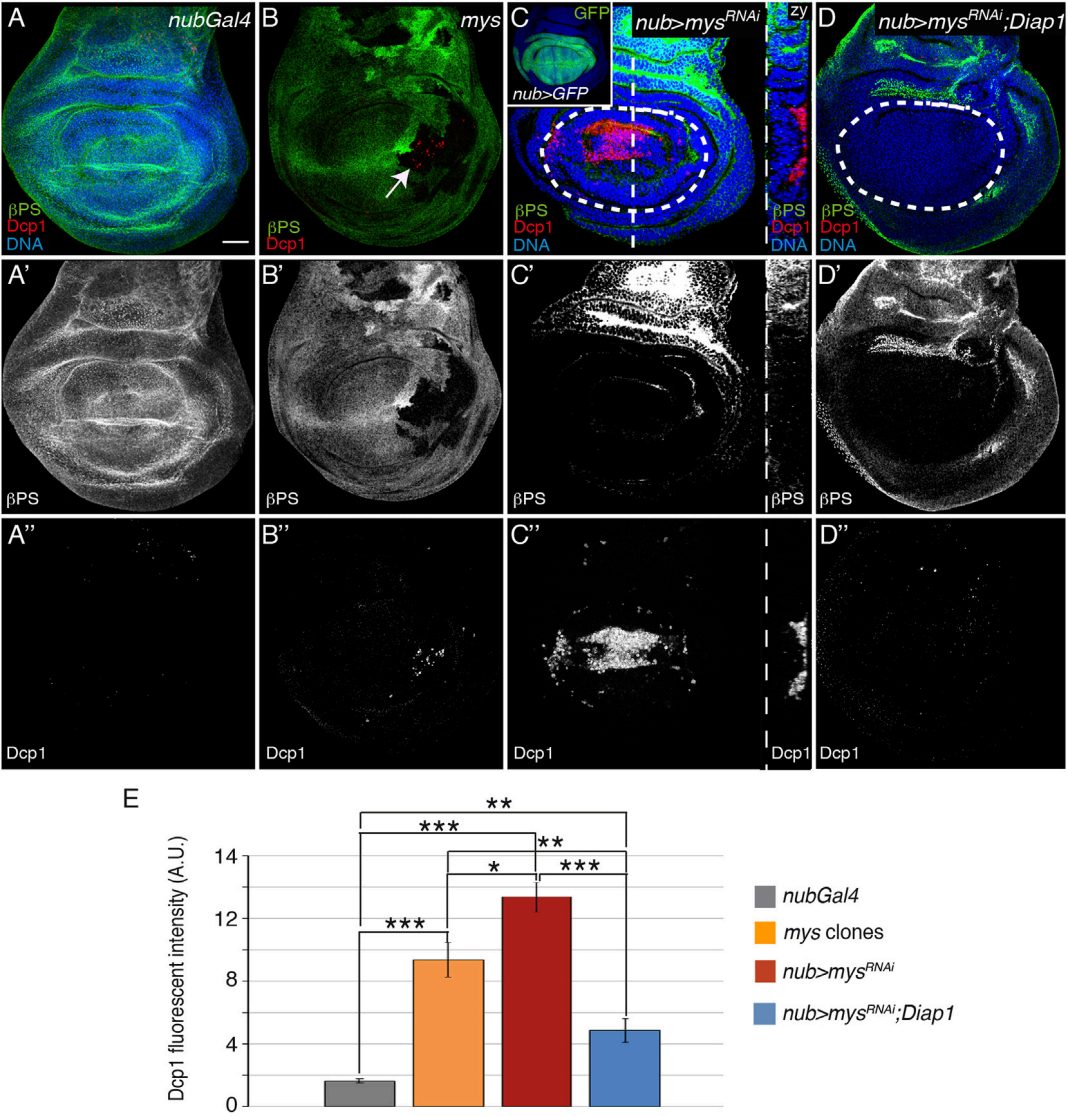
Integrins promote cell survival in the wing imaginal disc. **(A–D′′)** Maximal projection of confocal views of wing imaginal discs from third-instar larvae stained with anti-βPS [green in **(A–D)**, white in **(A′–D′)]**, anti-Dcp1 [red in **(A–D)**, white in **(A′′–D′′)]** and the nuclear marker Hoechst [DNA, blue in **(A–D)**]. **(A)** Control *nubGal4* wing disc. **(B)** Wing disc carrying *mys* mutant clones (white arrow), marked by the absence of βPS (green). **(C)** Wing disc expressing GFP or a *mys* RNAi under the control of *nubGal4*, *nub > GFP* (inset) and *nub > mys* ^*RNAi*^, respectively. (yz) Confocal *yz* section along the white dotted line shown in **(C)**. **(D)** Wing disc co-expressing a *mys* RNAi and Diap-1 under the control of *nubGal4*, *nub > mys* ^*RNAi*^; *Diap1*. **(E)** Quantification of Dcp1 levels in wing discs of the designated genotypes. The dotted white circles indicate the wing pouch area in all figures. The statistical significance of differences was assessed with a *t*-test, ***, ** and * *p* values < 0.001, <0.01, and <0.05, respectively. Scale bar in all panels, 30 μm.

To deepen our understanding of the role of integrins as survival factors during epithelia morphogenesis, we tested whether removing integrins in *Drosophila* wing disc epithelial cells affected their survival. The *Drosophila* genome contains two integrin β subunits, βPS and βν [reviewed in (Brown, 1993; Yee and Hynes, 1993). βPS, encoded by the gene *myospheroid* (*mys*), is the only β chain present in the wing imaginal disc (Brabant et al., 1996); Supplementary Figure S1]. First, we generated mosaic wing disc epithelia containing clones of cells homozygous for the null allele *mys* ^*XG43*^ (from now on *mys* cells, white arrow in Figures 1A–B”). To detect apoptosis, we used an antibody to cleaved effector caspase Dcp-1 (Yu et al., 2002). In control wing imaginal discs, Dcp-1 activity is very low, being detected only occasionally in a few cells scattered throughout the disc (Figure 1A, *n* = 20, where n represents the number of wing discs analyzed). In contrast, all wing discs containing *my*s clones showed apoptotic cells within the clone area (Figure 1B, *n* = 18). Second, we reduced integrin levels in larger areas of the disc using the *nubbin* (*nub*) Gal4 line to express a *mys* RNAi (*nub>mys RNAi*) in the wing pouch (inset in Figure 1C) region in the center of the disc that gives rise to the adult wing, indicated with a dotted white circle in all figures (Brand and Perrimon, 1993; Calleja et al., 1996). Immunostaining of control (*n* = 30) and experimental *nub>mys* ^*RNAi*^ wing imaginal discs (*n* = 36) with antibodies against the βPS protein and cleaved Dcp-1 showed that the expression of the *mys* specific RNAi in the wing pouch caused a strong reduction in βPS protein levels (Figures 1C,C’) and a robust increase in apoptosis (Figures 1C,C’’,E). Thus, integrin expression in wing disc epithelial cells prevent them from undergoing cell death. It is known that embryonic epithelial cells undergoing apoptosis are removed from the epithelium by extrusion rather than phagocytosis (Rosenblatt et al., 2001). Similarly, integrin-defective cells were extruded (Figures 1C–C’’) and accumulated between the basal surface of the disc epithelium and the ECM, as seen with an antibody against the ECM component Perlecan (Supplementary Figure S2).

Integrins can mediate both signaling and adhesion [reviewed in (Adams and Watt, 1993)]. To address which integrin function is required to facilitate cell survival in the wing imaginal disc, we used a chimeric integrin (diβ) that lacks integrin-adhesive function but maintains its ability to signal (Martin-Bermudo and Brown, 1999; Dominguez-Gimenez et al., 2007). We had previously shown that expression of diβ in the wing disc inhibited the basal localization of endogenous integrin [(Supplementary Figures S3B,B’; (Dominguez-Gimenez et al., 2007)]. Here, we found that it caused an increase in apoptosis (Supplementary Figures S3B,B”), strongly suggesting that integrin signaling is not sufficient to prevent cell death in detached wing disc cells.

Altogether, these results propose that integrin-mediated adhesion to the ECM promotes cell survival in the wing disc epithelium by regulating caspase activity. To further support this hypothesis, we tested whether overexpression of one of the *Drosophila* IAP (inhibitor of apoptosis) proteins, Diap1, suppressed the cell death caused by expression of *mys* RNAi and found that it did (Figures 1D-D’’,E, *n* = 35).

### Integrins DownRegulate Caspase Activity by Inhibiting *Hid* Expression

**FIGURE 2:**
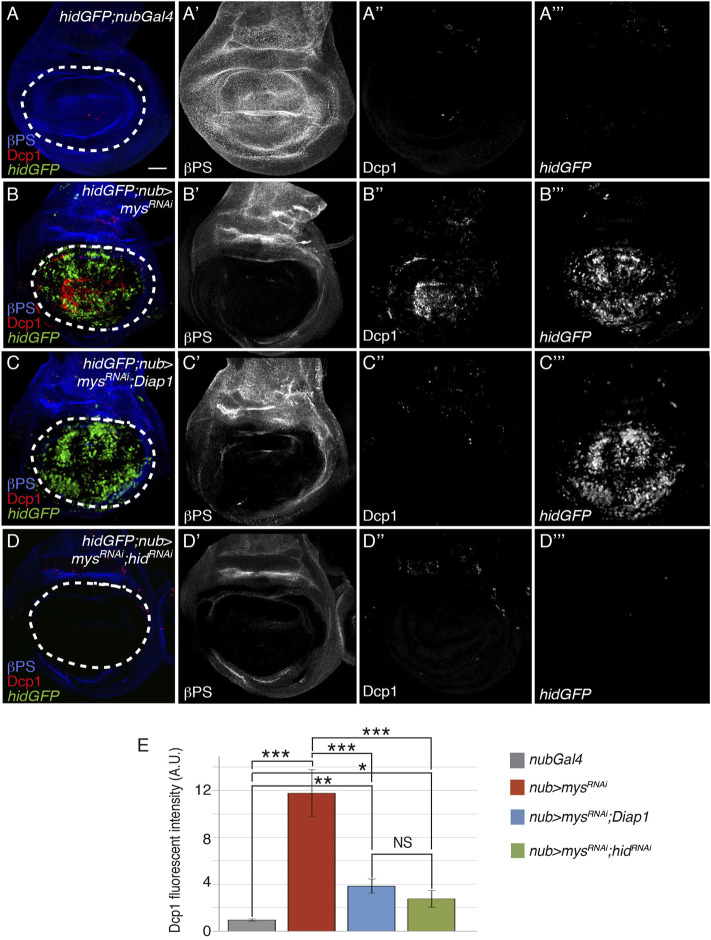
Integrins stimulate cell survival through inhibition of *hid* expression. **(A–D‴)** Maximal projection of confocal views of third-instar wing imaginal discs stained with anti-βPS [blue in **(A–D)**, white in **(A′–D′)]**, anti-Dcp1 [red in **(A–D)**, white in **(A′′–D′′)]** and anti-GFP [green in **(A–D)**, white in **(A‴–D‴)]**. **(A–A‴)** Control *nubGal4* wing disc carrying a reporter of *hid* expression, *hidGFP*, *hidGFP; nubGal4*. **(B–B‴)** *hidGFP* wing disc expressing a *mys* RNAi under the control of *nubGal4*, *hidGFP;nub > mys* ^*RNAi*^. **(C–D‴)** *hidGFP* wing disc co-expressing a *mys* RNAi under the control of *nubGal4* with either Diap1, *hidGFP; nub > mys* ^*RNAi*^; *Diap1* **(C–C‴)**, or a *hid* RNAi, *hidGFP;nub > mys* ^*RNAi*^; *hid* ^*RNAi*^ **(D–D‴)**. **(E)** Quantification of Dcp1 levels in wing discs of the indicated genotypes. The statistical significance of differences was assessed with a *t*-test, ***, ** and * *p* values < 0.001, <0.01, and <0.05, respectively. NS, non-statistically significant. Scale bar in all panels, 30 μm.

Previous experiments have shown that overexpression of *hid* or *rpr* induces downregulation of Diap1 levels and apoptosis in *Drosophila* wing imaginal discs (Milan et al., 1997; Bergantinos et al., 2010; Yoo et al., 2002). As Diap1 overexpression rescues cell death due to integrin downregulation, integrins could promote cell survival through the regulation of *hid* and/or *rpr* expression. To test this, we analysed *hid* and *rpr* expression in control and *nub>mys* ^RNAi^ wing discs, using the *hid* 5′F-GFP [from now on *hidGFP*, (Tanaka-Matakatsu et al., 2009)] and *rprLacZ* (Nordstrom et al., 1996) transcriptional reporters. *hidGFP* levels in control discs (*hidGFP*; *nubGal4*) are hardly detectable, consistent with a low occurrence of cell death in this tissue (Tanaka-Matakatsu et al., 2009); Figures 2A-A’’’,E]. This expression was dramatically upregulated in *nub > mys* ^*RNAi*^ discs (*n* = 25, Figures 2B-B’’’). In contrast, the normal expression pattern of *rprLacZ*, confined to a small region of the notum and two stripes in the D-V and A-P boundaries [(Nordstrom et al., 1996), Supplementary Figures S4A,A’], was not altered in *nub > mys* ^RNAi^ discs (*n* = 28, Supplementary Figures S4B,B’). These results suggest that integrins block *hid*, but not *rpr* expression in normal wing disc epithelial cells to promote cell survival. Furthermore, consistent with the idea that *Drosophila hid* acts upstream of *Diap1* (Wang et al., 1999; Goyal et al., 2000), we found that expression of Diap1 was not able to rescue the increase in *hidGFP* levels observed in *mys* ^RNAi^ cells (*n* = 24, Figures 2C–C’’’).

To further confirm *hid* involvement in integrin loss of function-dependent cell death, we tested whether reducing *hid* levels could rescue apoptosis in *nub > mys* ^*RNAi*^ discs. We found that expression of an RNAi against *hid* (*hidGFP*; *nub > mys* ^*RNAi*^; *hid* ^*RNAi*^) resulted in a significant reduction of *mys RNAi*-induced apoptosis (*n* = 20, Figures 2A-A’’’, B-B’’’, D-D’’’, E). These findings demonstrate that integrins promote cell survival in wing imaginal discs through the negative regulation of *hid* expression.

Altering the levels of Decapentaplegic (Dpp), a *Drosophila* transforming growth factor β homologue), in wing discs results in upregulation of brinker (*brk*), a transcriptional repressor that prevents apoptosis (Adachi-Yamada et al., 1999; Moreno et al., 2002). However, reducing integrin function does not seem to affect Dpp signalling, as the expression pattern of the *brk* reporter *brkLacZ* was not altered in *nub > mys* ^RNAi^ wing discs (*n* = 26, Supplementary Figure S5B).

### Downregulation of Integrin Expression in Wing Discs Induces JNK Activation

**FIGURE 3:**
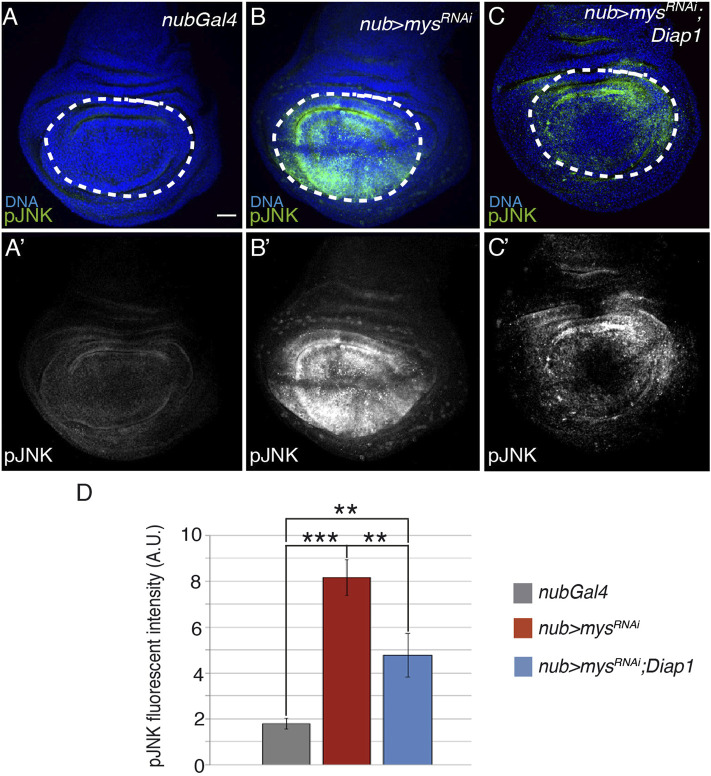
Elimination of integrins activates the JNK pathway. **(A–C′)** Maximal projection of confocal views of wing imaginal discs from third-instar larvae stained with anti-pJNK [green in **(A–C)**, white in **(A′–C′)]** and the nuclear marker Hoechst [DNA, blue in **(A–C)**]. **(A,A′)** Control *nubGal4* wing disc. **(B,B′)** Wing disc expressing a *mys* RNAi under the control of *nubGal4*, *nub > mys* ^*RNAi*^. **(C,C′)** Wing disc co-expressing RNAi against *mys* and *Diap1* under the control of *nubGal4*, *nub > mys* ^*RNAi*^ *; Diap1*. **(D)** Quantification of pJNK levels in wing discs of the indicated genotypes. The statistical significance of differences was assessed with a *t*-test, *** and ** *p* values < 0.001 and <0.01, respectively. Scale bar in all panels, 30 μm.

As mentioned in the introduction, the role of JNK in anoikis is controversial, as, depending on the cell type and cellular context, it can be either essential (Frisch et al., 1996) or dispensable (Khwaja and Downward, 1997). In *Drosophila* wing discs, JNK activation triggers apoptosis in response to several stimuli, including distortion of positional information (Adachi-Yamada et al., 1999), TNF signalling (Igaki et al., 2002; Moreno et al., 2002) or irradiation (Luo et al., 2007). To study the role of the JNK pathway in apoptosis induced by integrin loss of function, we analyzed JNK activation using an antibody against phosphorylated JNK (pJNK, Figure 3). Compared to controls (*n* = 16, Figures 3A,A’,D), pJNK levels were upregulated in *nub > mys* ^RNAi^ discs (*n* = 18, Figures 3B,B’,D), suggesting that integrins control JNK activity in the wing disc. This was further confirmed using a reporter that monitors the transcription of a target of the JNK pathway, the JNK inhibitor *puckered* (*pucLacZ*) (Adachi-Yamada et al., 1999). We found that while in wild type wing discs, *pucLacZ* expression was restricted to a small proximal region (Adachi-Yamada et al., 1999), Supplementary Figures S6A,A’), in *nub > mys* ^RNAi^ wing discs, *pucLacZ* levels were strongly upregulated in the pouch (*n* = 28,Supplementary Figures S6B,B’).

In some contexts, JNK signalling can be activated as a consequence of apoptosis (Kuranaga et al., 2002). To test whether activation of JNK lies upstream or downstream of loss of integrin function, JNK activation was assessed in *nub > mys* ^*RNAi*^; *Diap1* wing discs (Figure 3). We found that pJNK (*n* = 20, Figures 3C,D) and *pucLacZ* (*n* = 25, Supplementary Figures S6C,C’) levels were still upregulated despite rescue of apoptosis (Figure 1D), suggesting that JNK activation is upstream of cell death due to integrin loss of function in wing disc cells. Stress induced cell death in the wing disc activates JNK upstream of caspase activator proteins, which, in turn, reinforce JNK activation in a positive feedback loop to amplify the apoptotic response (Shlevkov and Morata, 2012). Here, we found that, in fact, the increase in JNK activity of *nub > mys* ^*RNAi*^; *Diap1* discs was lower than the one observed in *nub > mys* ^*RNAi*^ discs (Figures 3B–D). This result suggests that the feedback loop between JNK activity and apoptosis observed in stress-induced apoptosis may also operate in integrin loss of function-dependent apoptosis.

### JNK Mediates Apoptosis due to Loss of Integrin Function in Wing Disc Cells

**FIGURE 4:**
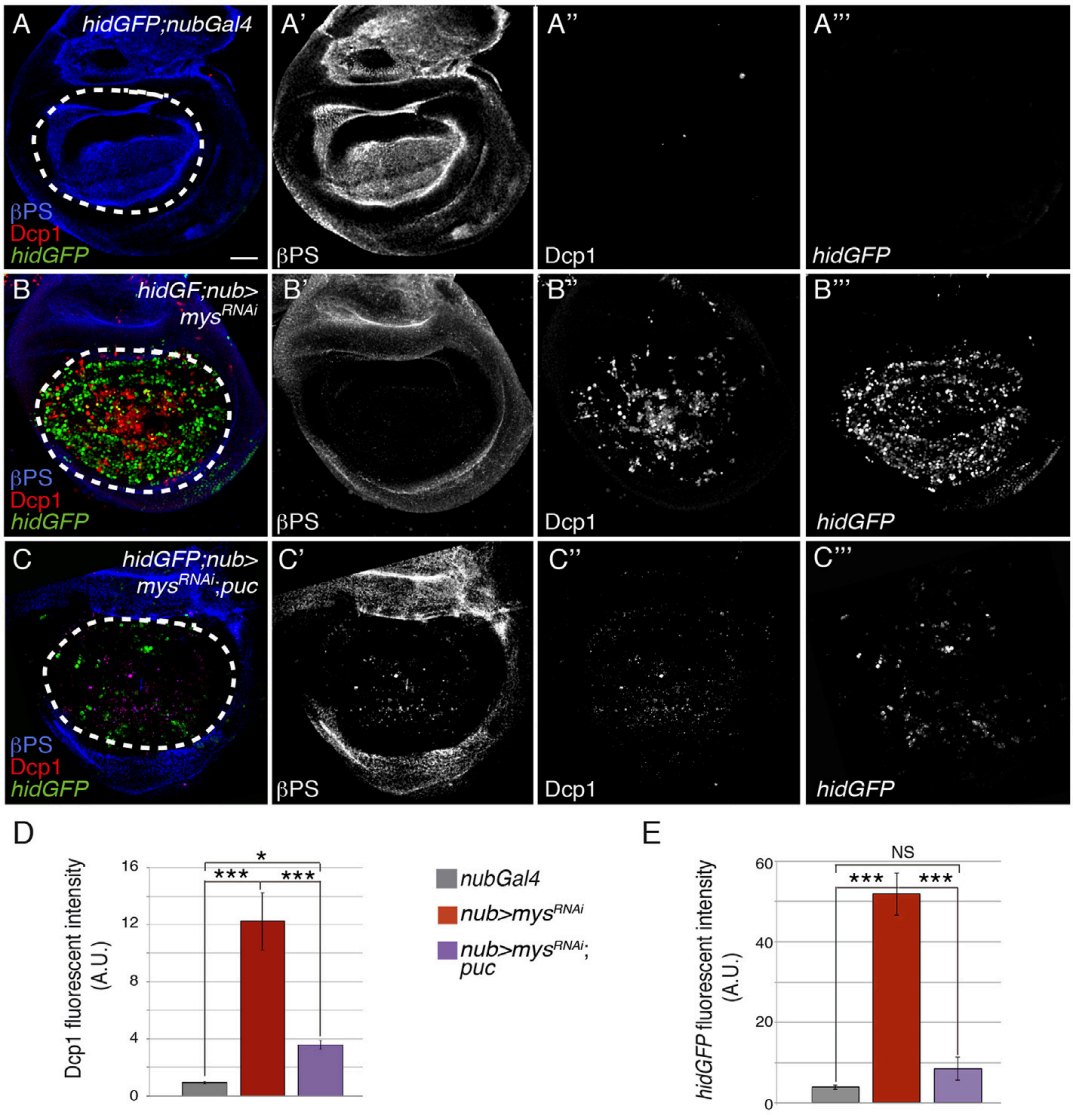
Integrins promote cell survival by inhibiting the JNK pathway. **(A–C‴)** Maximal projection of confocal views of third-instar wing imaginal discs carrying the reporter *hidGFP*, stained with anti-βPS [blue in **(A–C)**, white in **(A′–C′)]**, anti-Dcp1 [red in **(A–C)**, white in **(A′′–C′′)]** and anti-GFP [green in **(A–C)**, white in **(A‴–C‴)]**. **(A–A‴)** Control *hidGFP*; *nubGal4* wing disc. **(B–B‴)** *hidGFP* wing disc expressing a *mys* RNAi under the control of *nubGal4*, *hidGFP*; *nub > mys* ^*RNAi*^. **(C–C‴)** *hidGFP* wing disc co-expressing a *mys* RNAi and the negative regulator of the JNK pathway *puc*, under the control of *nubGal4 hidGFP*; *nub > mys* ^*RNAi*^; *puc*. **(D,E)** Quantification of Dcp1 **(D)** and *hidGFP* **(E)** levels in wing discs of the indicated genotypes. The statistical significance of differences was assessed with a *t*-test, *** and * *p* values < 0.001, <0.01, and <0.05, respectively. NS, non-statistically significant. Scale bar in all panels, 30 μm.

To test whether the JNK pathway mediates cell death due to integrin loss of function in wing imaginal discs, we analyzed if overexpression of the JNK inhibitor *puc* suppressed the cell death caused by *mys* RNAi (*nub > mys* ^*RNAi*^ *;puc*) and found that it did (Figures 4A–D, *n* = 24).

The relationship between JNK signalling, pro-apoptotic proteins and caspase activation is context-dependent. Thus, the JNK pathway can act upstream or downstream of pro-apoptotic genes such as *rpr* or *hid*. For instance, experiments in *Drosophila* S2 cells suggested that Rpr could stimulate JNK activation through DIAP1 degradation (Kuranaga et al., 2002). In contrast, JNK signalling promotes *hid* expression (and apoptosis) in eye discs, and it is required for *rpr*-reporter induction in response to radiation in wing discs (Kuranaga et al., 2002; Moreno et al., 2002). In our system, inhibition of JNK signalling *via puc* overexpression was able to suppress *hid* upregulation in *nub > mys* ^*RNAi*^ wing discs to a large extent (*n* = 17, Figures 4C-C’’’,E). Altogether, these results allow us to conclude that reduction of integrin function in wing discs leads to apoptosis by stimulating *hid* transcription through JNK activation.

### Integrins Cooperate With the EGFR Pathway to Promote Cell Survival

**FIGURE 5:**
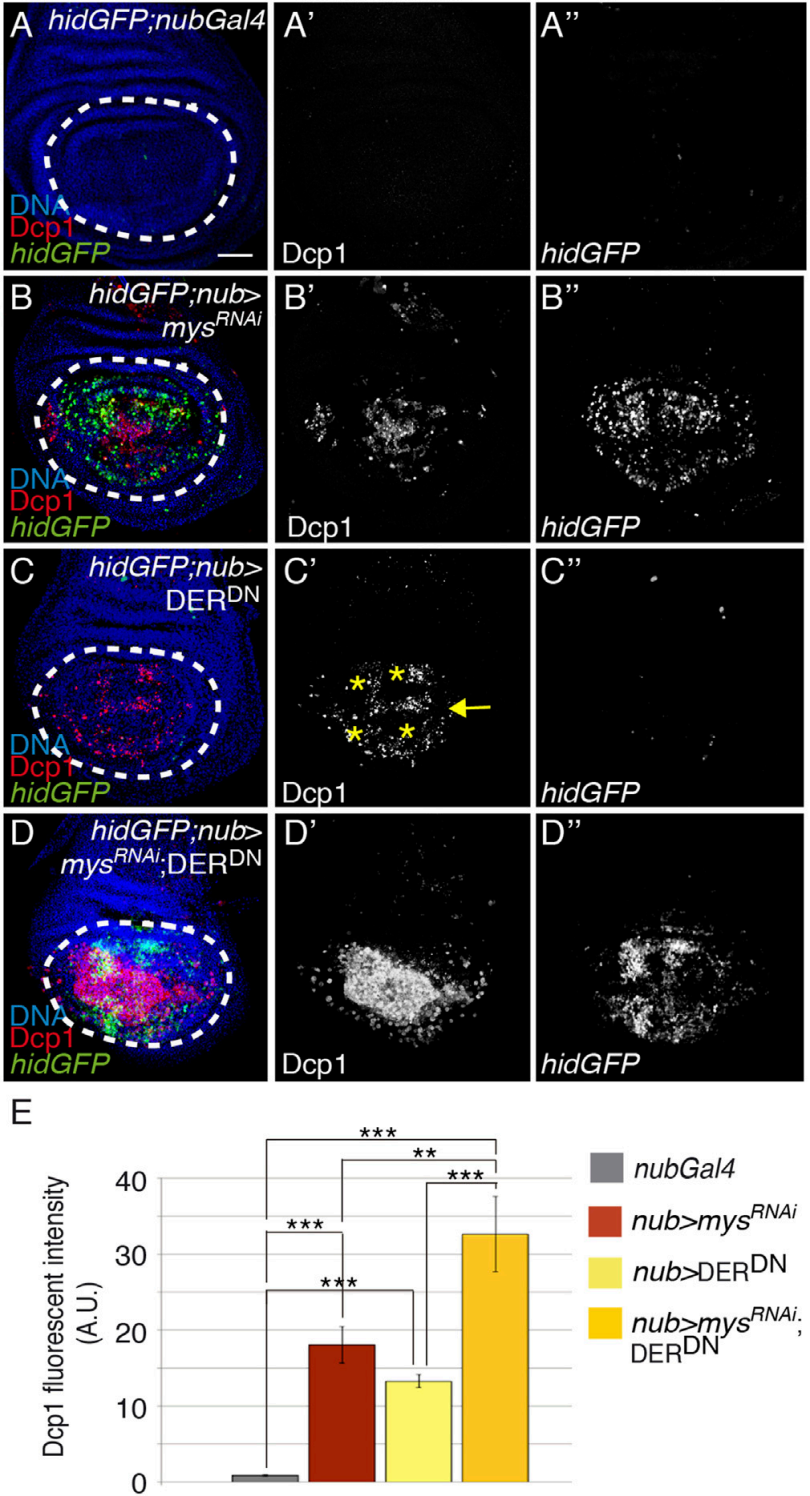
Integrins and EGFR cooperate to promote cell survival. **(A–D”)** Maximal projection of confocal views of wing imaginal discs from third-instar larvae carrying the reporter *hidGFP*, stained with anti-Dcp1 [red in **(A–D)**, white in **(A′–D′)]**, anti-GFP [green in **(A–D)**, white in **(A’’–D’’)]** and the nuclear marker Hoechst [DNA, blue in **(A–D)**]. **(A–D’’)** Control *hidGFP*; *nubGal4* wing disc. **(B–B’’)** *hidGFP* wing disc expressing a *mys* RNAi under the control of *nubGal4*, *hidGFP;nub > mys* ^*RNAi*^. **(C–C’’)** *hidGFP* wing disc expressing a dominant negative form of the EGFR, DER^DN^, under the control of *nubGal4*, *hidGFP*; *nub >* DER^DN^. **(C–D’’)** *hidGFP* wing disc co-expressing a *mys* RNAi and DER^DN^ under the control of *nubGal4 hidGFP*; *nub > mys* ^*RNAi*^; DER^DN^. **(E)** Quantification of Dcp1 levels in wing discs of the indicated genotypes. The statistical significance of differences was assessed with a *t*-test, *** and ** *p* values < 0.001 and <0.01, respectively. NS, non-statistically significant. Scale bar in all panels, 30 μm.

The EGFR activates a network of signaling pathways promoting survival in many different cellular contexts [reviewed in (Henson and Gibson, 2006)]. In addition, experiments mainly from cell culture have revealed synergistic cooperation between growth factors and integrins in cell survival (Reginato et al., 2003; Miranti and Brugge, 2002)*.* For instance, EGFR promotes survival through regulation of the Ras/MAPK pathway and consequent inhibition of the proapoptotic gene *hid* in the eye disc (Bergmann et al., 1998; Kurada and White, 1998; Yu et al., 2002). Moreover, wing disc mutant clones for EGFR or for some of their dowmstream effectors, such as Ras, are smaller than their twin spot, suggesting a role for the EGFR pathway in cell survival (Diaz-Benjumea and Garcia-Bellido, 1990; Diaz-Benjumea and Hafen, 1994; Zecca and Struhl, 2002). To directly address a role for EGFR in cell survival in the wing disc, we expressed an EGFR dominant negative construct (UAS-DER^DN^) under the control of the nub-Gal4 line and found it led to increase cell death in the pouch region (*nub >* DER^DN^, *n* = 21, Figures 5A, A’, C, C’, E). However, in contrast to what happens in *nub > mys* ^RNAi^ wing discs (Figures 5B, B’, E), where cell death was found distributed all over the pouch, expression of DER^DN^ restricted apoptosis to regions of high EGFR activity (Gabay et al., 1997), such as the wing margin and the presumptive vein territories (yellow arrow and asterisks in Figures 5A, A’, C, C, respectively). In addition, and contrary to what happens in the eye, EGFR did not seem to exert its pro-survival function through regulation of *hid* or *rpr* transcription, as *hidGFP* or *rpr*-lacZ expression were not altered in *nub >* DER^DN^ wing discs (Figures 5A, A’, C, C”; Supplementary Figure S7). We thus conclude that the EGFR pathway has a limited role in the control of cell survival in the wing disc that is independent of the transcriptional regulation of the pro-apoptotic genes *hid* and *rpr*. Finally, we examined a possible cooperation between integrins and the EGFR pathway to mediate cell survival. We found that the simultaneous co-expression of *mys* ^*RNAi*^ and DER^DN^ in wing discs (*nub > mys* ^*RNAi*^; DER^DN^) enhanced the cell death phenotype caused by the expression of any of them on their own (Figures 5B–E), strongly suggesting that integrins and the EGFR pathway act in paralell to promote cell survival in wing imaginal discs.

### Oncogenic Ras Suppresses Anoikis in Wing Discs

**FIGURE 6:**
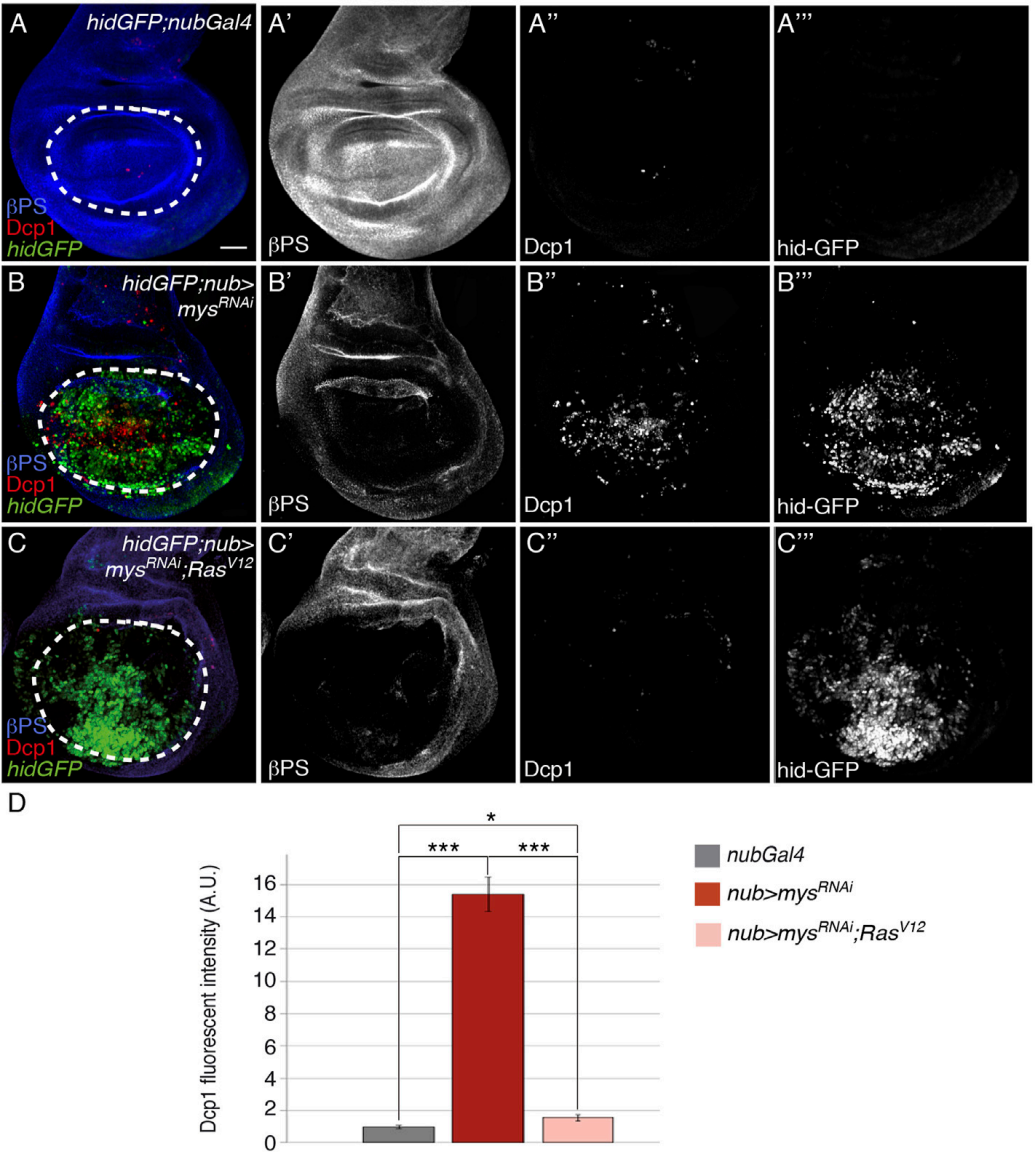
Ectopic activation of the Ras pathway rescues anoikis. **(A–C’’’)** Maximal projection of confocal views of third-instar wing imaginal discs carrying the reporter *hidGFP*, stained with anti-βPS [blue in **(A–C)**, white in **(A′–C′)]**, anti-Dcp1 [red in **(A–C)**, white in **(A’’–C’’)]** and anti-GFP [green in **(A–C)**, white in **(A‴–C‴)]**. **(A–A‴)** Control *hidGFP;nubGal4* wing disc. **(B–B‴)** *hidGFP* wing disc expressing a *mys* RNAi under the control of *nubGal4*, *hidGFP*; *nub > mys* ^*RNAi*^. **(C–C‴)** *hidGFP* wing disc co-expressing a *mys* RNAi and an activated form of Ras, Ras^V12^, under the control of *nubGal4 hidGFP*; *nub > mys* ^*RNAi*^; Ras^V12^. **(D)** Quantification of Dcp1 levels in wing discs of the indicated genotypes. The statistical significance of differences was assessed with a *t*-test, *** and * *p* values < 0.001 and <0.05, respectively. Scale bar in all panels, 30 μm.

Suppression of anoikis after detachment of cancer cells from the ECM is a key step in tumor metastasis [reviewed in (Simpson et al., 2008)]. In fact, the ability of oncogenic Ras to suppress anoikis has long been considered critical to Ras transformation. However, recent evidence suggests that Ras and other oncogenes can induce both pro- and anti-apoptotic signals depending on the cell type and context [reviewed in (Cox and Der, 2003)]. In *Drosophila*, Ras overactivation renders cells refractory to stress- and irradiation-induced apoptosis (Bergmann et al., 1998; Kurada and White, 1998; Pinal et al., 2018). To test whether oncogenic Ras could protect cells from anoikis in the wing imaginal disc, we overexpressed an activated form of DRas, Ras^V12^ (Karim and Rubin, 1998) and found it substantially suppressed the cell death phenotype caused by integrin depletion (*nub > mys* ^RNAi^, *ras* ^V12^; Figures 6A-A’’, 6B–B’’, C–C’’, D, *n* = 18). These results demonstrate that oncogenic Ras is able to suppress anoikis in wing imaginal discs. In epithelial cell lines, oncogenic Ras confers resistance to anoikis partly by downregulating the expression of proapoptotic Bcl-2 members (Rak et al., 1995) and by preventing downregulation of antiapoptotic proteins (Rosen et al., 2000). In *Drosophila*, it appears that the Ras pathway regulates Hid activity at the level of both, protein phosphorylation and gene expression, in *hid*-induced apoptosis (Bergmann et al., 1998; Kurada and White, 1998). However, we found that, in the *Drosophila* wing disc epithelium, the ability of oncogenic Ras to confer resistance to anoikis does not involve transcriptional regulation of *hid*, as *hidGFP* levels were not altered in *nub > mys* ^RNAi^; Ras^V12^ (*n* = 21, Figures 6A’’’, C’’’) discs compared to *nub > mys* ^RNAi^ (*n* = 20, Figure 6B’’’).

**FIGURE 7:**
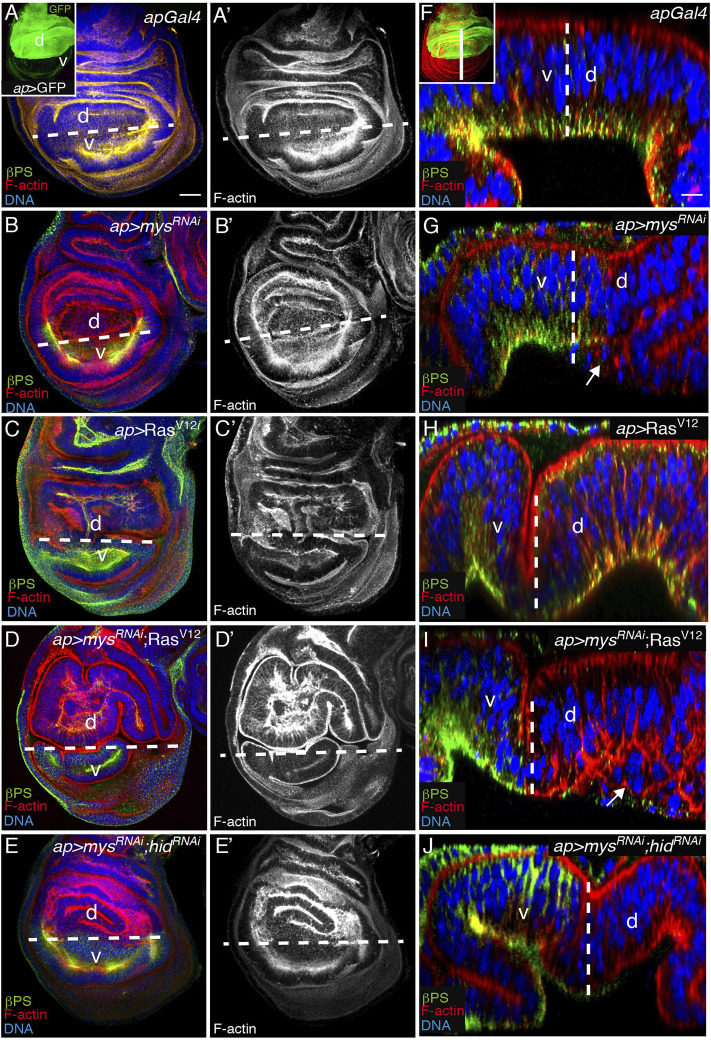
Integrin knockdown stimulates basal extrusion of Ras^V12^ tumor cells. **(A–J)** Wing imaginal discs from third-instar larvae expressing the indicated UAS transgenes under the control of *ap*-Gal4 (inset in A), stained with anti-βPS [green in **(A–E)** and **(F–J)]**, the nuclear marker Hoechst [DNA, blue in **(A–E,F–J)**] and Rhodamine Phalloidin to detect F-actin [red in **(A–E)** and **(F–J)** and white in **(A′–E′)]**. **(A–E′)** Maximal projection of confocal views of wing imaginal discs. **(A,A′)** Control *apGal4* wing disc. **(B,B′)** Wing disc expressing a *mys* RNAi under the control of *apGal4*, *ap > mys* ^*RNAi*^. **(C,C′)** Wing disc expressing Ras^V12^ under the control of *apGal4*, *ap >* Ras^V12^. **(D,D′)** Wing disc co-expressing *mys* RNAi and Ras^V12^ under the control of *apGal4*, *ap > mys* ^*RNAi*^; Ras^V12^. **(E–E′)** Wing disc co-expressing RNAis against *mys* and *hid* under the control of *apGal4*, *ap > mys* ^*RNAi*^; *hid* ^*RNAi*^. **(F–J)** Confocal *yz* sections along the white straight line shown in inset in F of wing discs of the indicated genotypes. White dotted lines in all panels separate dorsal (d) from ventral (v) compartments. White arrows in G and I point to dead **(G)** and alive **(I)** cells extruded from the wing disc epithelium. Scale bar 30 μm **(A–E′)** and 10 μm **(F–J)**.

Ectopic expression of Ras^V12^ in the dorsal compartment of wing imaginal discs, by means of the *apterous* Gal4 (*ap*) Gal4 line, causes tissue overgrowth and cell shape changes, which results in the formation of ectopic folds [*ap* > Ras^V12^, *n* = 20, Figures 7A,A’,C,C’, (Prober and Edgar, 2000; Soler Beatty et al., 2021). Reducing integrin function also alters cell shape causing a mild folding phenotype (Dominguez-Gimenez et al., 2007), Figures 7B, B’ E, E’]. Here, we found that co-expression of *mys* ^*RNAi*^ and Ras^V12^ enhanced the cell shape and folding phenotype caused by the sole expression of any of them (*ap* > *mys* ^*RNAi*^; Ras^V12^, *n* = 20, Figures 7D,D’).

In this work, we show that reducing integrin expression ensued basal extrusion of the dead cells (Figures 1C–C’’, Figures 7F,G). However, even though oncogenic K-Ras MCDK cells survive and extrude basally, a mechanism proposed to initiate invasion into surrounding tissues (Slattum et al., 2014), we found that expression of Ras^V12^ on its own in wing disc cells (*ap* > Ras^V12^) did not result in cell extrusion (Figure 7H). In striking contrast, the expression of Ras^V12^ in integrin mutant cells not only allowed their survival (Figure 6) but also induced their basal extrusion beneath the surface of the wing disc epithelia (*ap* > *mys* ^*RNAi*^; Ras^V12^, *n* = 20, Figure 7I). Furthermore, because depletion of both integrins and *hid* (*ap* > *mys* ^*RNAi*^; *hid* ^*RNAi*^) did not result in cell extrusion (Figure 7J), the basal extrusion of *mys*; Ras^V12^ wing disc cells was not due only to the ability of Ras^V12^ to rescue apoptosis. From the above results we conclude that integrins prevent extrusion of transformed Ras^V12^ cells.

## Discussion

Epithelial cells monitor their environment looking for signals necessary for survival. Among those, attachment to the ECM is critical, as the dominant response of epithelial cells to anchorage loss is anoikis. However, at certain stages of development and during tissue repair epithelial cells survive in an unanchored state. Likewise, cellular transformation is accompanied by an inappropriate anoikis resistance, suggesting that anoikis can be modulated. Molecular mechanisms regulating anoikis have been described in several epithelial cell types reviewed in (Zhan et al., 2004). The relevance of these mechanisms in epithelial cells in a physiological setting is unclear, as most of these studies use immortalized or cancer cell lines. Here, we show that integrins act as survival factors during the development of the epithelial wing disc and that the EGFR/Ras pathway protects epithelial cells from anoikis both in developmental and tumorigenic contexts.

Studies using standard 2D cultures and 3D matrices have revealed a central role for cell-ECM interactions -mostly mediated by integrins- in survival (Stupack and Cheresh, 2002). However, there is still little evidence up to date for a role for integrins as survival factors during tissue morphogenesis. The use of conditional knockouts in mice has revealed that the function of β1 integrins in developmental cell survival is complex and cell type- and differentiation state- specific. Thus, while β1 integrins are required for the survival of cranial cells of the hyoid arch region (Goh et al., 1997), granular cells of the kidney (Mohamed et al., 2020), lens cells (Simirskii et al., 2007), endodermal cells (Carlson et al., 2008) or the epiblast (Mole et al., 2021), it is dispensable for the survival of intestinal epithelial cells (Jones et al., 2006; Benoit et al., 2010). These differences in the contribution of murine β1 integrins to cell survival could depend on the repertoire of integrins expressed in each cell type and on a possible redundancy among them. Hence, the use of simpler model organisms with a markedly smaller number of integrins in their genome should help determine the real role of integrins in cell survival during development. However, there is no clear evidence to date of a function for integrins in cell survival in worms or flies. Our work demonstrates for the first time that integrins are required for epithelial cell survival in wing imaginal discs. We also show that integrins prevent anoikis by blocking the expression of IAP protein antagonists such as Hid. This is in contrast to vertebrate integrins, which seem to control anoikis mainly by controlling the expression and/or activation of anti- and pro-apoptotic Bcl-2 family proteins, known regulators of mitochondrial outer membrane potential and permeability (Stupack and Cheresh, 2002; Scorrano and Korsmeyer, 2003). In fact, mitochondria play crucial roles in the modulation of the anoikis response in mammals [reviewed in (Zhan et al., 2004)]. *Drosophila* contains two Bcl-2 related proteins, Debcl and Buffy, and one homolog of the mammalian mitochondrial serin protease HtrA2/Omi, which act as pro- or anti-apoptotic factors in developmental programmed cell death and *γ* irradiation-induced apoptosis (Brachmann et al., 2000; Colussi et al., 2000; Igaki et al., 2000; Quinn et al., 2003). These studies suggest that the mitochondrial regulation of cell death machinery could be conserved in *Drosophila* (Challa et al., 2007; Igaki et al., 2007). Future experiments are required to test whether mitochondria play any role in the regulation of anoikis in *Drosophila*, as it is the case in mammals.

Integrins are non-kinase receptors. Consequently, they require kinases to enact signaling pathways blocking apoptosis. The JNK pathway is among the earliest pathways activated upon detachment from the ECM in several systems [reviewed in (Liu and Lin, 2005)]. Yet, the role of JNK in anoikis is controversial, as it can be essential (Frisch et al., 1996) but also dispensable (Khwaja and Downward, 1997). In *Drosophila*, JNK signalling correlates with amnioserosa cell death during embryonic dorsal closure (Reed et al., 2004). As the amnioserosa undergoes premature disintegration in a proportion of integrin mutant embryos, it has been hypothesized that integrins block JNK-dependent anoikis in amnioserosa cells. However, a definitive proof of integrin depletion-dependent anoikis is still lacking. Our results show that JNK signaling is activated and required for anoikis in wing disc epithelial cells. In mammary epithelial cells, JNK stimulate anoikis by regulating the expression or activity of the mitochondrial regulator proteins Bcl-2 (Lei and Davis, 2003; Hubner et al., 2008; Girnius and Davis, 2017). In contrast, here, we find that JNK induces anoikis by promoting the expression of Hid in the *Drosophila* wing imaginal disc. The pro-apoptotic role of JNK by modulating the expression of antagonists of IAP proteins is not exclusive to anoikis, as it also occurs in response to radiation in wing discs (Pinal et al., 2018) and to loss of apical determinants in the embryonic epidermis (Kolahgar et al., 2011). A role for the *Drosophila* JNK pathway on inducing any type of cell death through mitochondrial regulation awaits further investigations.

**FIGURE 8:**
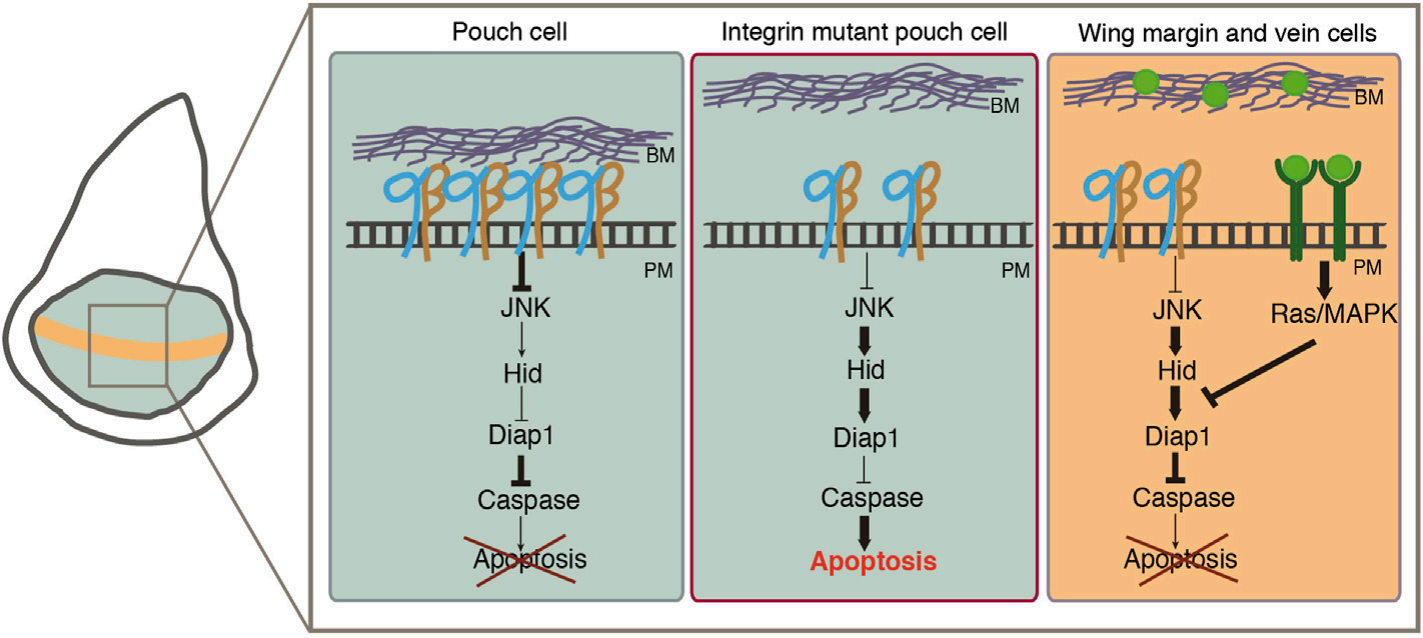
Model showing how integrins and the EGFR pathway act together to promote cell survival in wing imaginal disc cells. On the left, schematic representation of a wing disc with the wing pouch colored in green and the wing margin in orange. On the right, proposed molecular mechanism by which integrins regulate cell survival. (Left rectangle) In pouch wing imaginal disc cells, integrin-mediated adhesion to the ECM regulates cell survival by repressing the expression of the pro-apoptotic gene *hid via* inhibition of JNK activity. (Middle rectangle) In the absence of integrins JNK activity levels increase leading to high levels of Hid expression, caspase activation and apoptosis. (Right rectangle) The EGFR (green) pathway prevents the death of wing margin and vein cells when they detach from the ECM during wing morphogenesis.

Extracellular growth factors are required for the survival of most animal cells. Growth factors regulate cell survival by stimulating the antiapoptotic activity of the EGFR/Ras pathway. In the *Drosophila* embryo, induction of Ras leads to downregulation of *hid* mRNA levels (Kurada and White, 1998). In the eye disc, ectopic expression of either an inhibitor of the EGFR or a dominant negative form of the receptor results in apoptosis (Freeman, 1996; Sawamoto et al., 1998). Here, we show that the activity of the EGFR is required in the wing disc epithelium for the survival of cells exhibiting high activity of the pathway, such as the wing margin and future vein cells. During morphogenesis, epithelial cells need to detach from the ECM to allow changes in cell and tissue shape. In this context, epithelial cells are required to survive in an unanchored state. This is the case of the wing margin cells, which, at the onset of pupariation, detach from the basement membrane and shorten to drive bending of the wing pouch and formation of the two bi-layer adult wing (Fristrom and Fristrom, 1993). Similarly, vein formation also involves changes in cell shape and adhesion to the underlying basement membrane (De Celis, 2003). We propose that the EGFR pathway protects vein and wing margin cells from anoikis during wing morphogenesis (Figure 8).

Oncogenic Ras has been shown to suppress caspases function in several systems (Cox and Der, 2003). In the *Drosophila* eye disc, oncogenic Ras blocks *hid*-induced apoptosis by inhibiting Hid phosphorylation (Bergmann et al., 1998). Here, we show that oncogenic Ras rescues loss of integrin-dependent cell death in the wing disc without decreasing *hid* transcription levels. These results suggest that the mechanisms by which oncogenic Ras confers resistance to cell death in *Drosophila* might be cell and stimuli specific. One prominent feature of malignant cells is the presence of altered cell shape and cytoskeleton, which contributes to increase resistance to apoptosis and neoplastic growth (Martin and Leder, 2001). Our results show that, besides blocking cell death, expression of Ras^V12^ enhances the cell shape phenotype due to integrin donwregulation. In the future, it will be interesting to analyse the contribution of aberrant cell shape to anoikis resistance in transformed epithelial wing disc cells.

Finally, cancer cells can spread by extrusion-initiated programs that couple anoikis resistance with cell extrusion. Furthermore, basolateral extrusion is generally thought to promote spread and invasion of tumor cells and, eventually, formation of metastatic lesions (Jiang et al., 2015). However, the mechanisms controlling basal extrusion of tumor cells are still unclear. Here, we show that elimination of integrin function results on basal extrusion of Ras^V12^ cells. We can now use the advantages of the *Drosophila* model system to increase our understanding of the mechanisms by which downregulation of integrin function induces basal extrusion of cancer cells.

In summary, our results reveal that integrins act as survival factors in the wing disc epithelium. We also demonstrate that integrins cooperate with the EGFR pathway to prevent anoikis during wing disc morphogenesis (Figure 8). Finally, we show that oncogenic activation of the Ras pathway results in anoikis resistance and basal cell extrusion. The identification of the molecular and cellular mechanisms by which integrins act as survival factors will help us to understand not only morphogenesis but also cancer progression.

## Data Availability

The raw data supporting the conclusion of this article will be made available by the authors, without undue reservation.
