# Supplementary figures and images for: Integrins Cooperate With the EGFR/Ras Pathway to Preserve Epithelia Survival and Architecture in Development and Oncogenesis
Source: Front Cell Dev Biol. 2022 Jun 13;10:892691. doi: 10.3389/fcell.2022.892691 (PMC9234701; doi:10.3389/fcell.2022.892691)

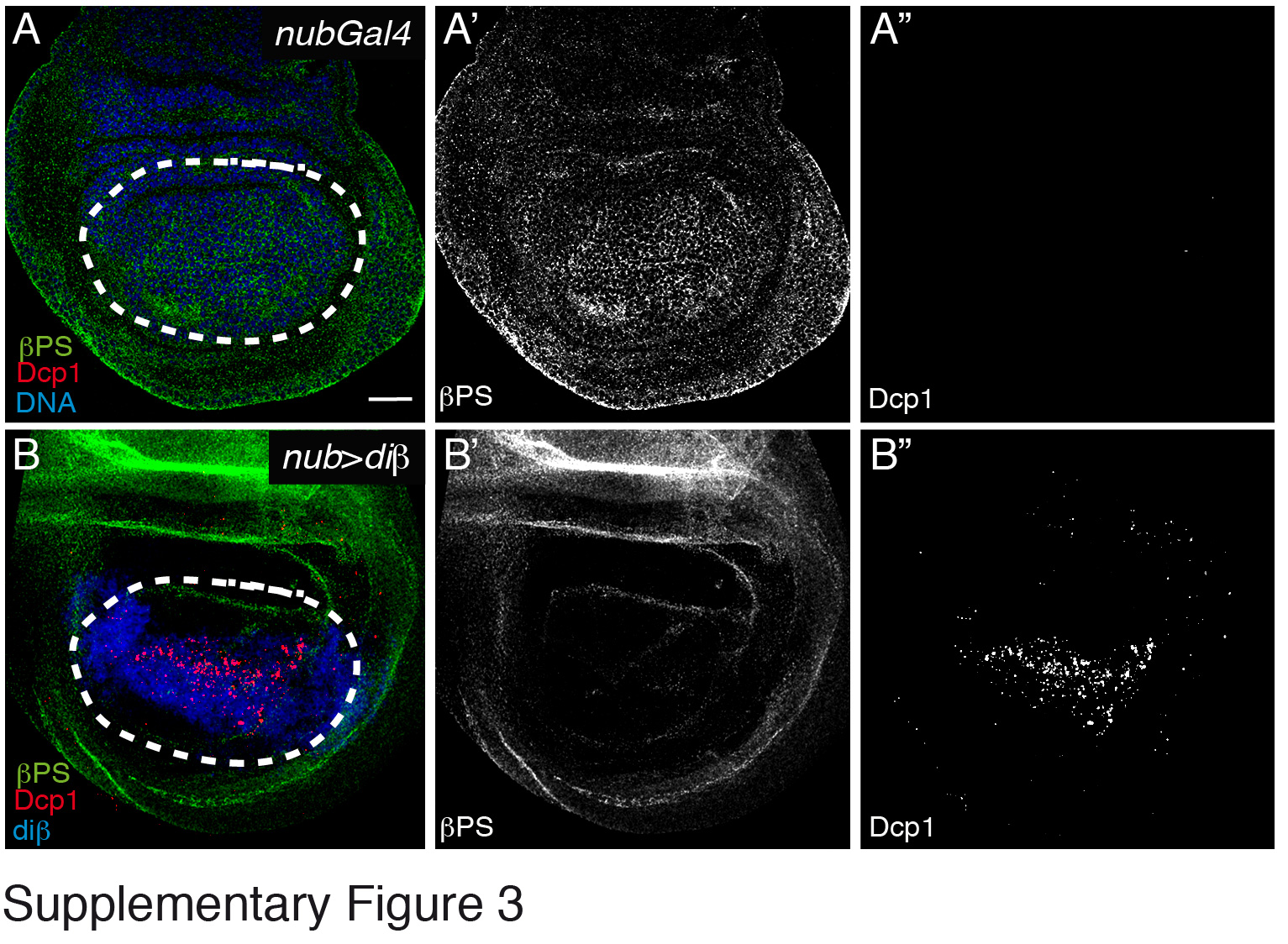

Supplement: Supplementary file 1 [file Image3.JPEG]

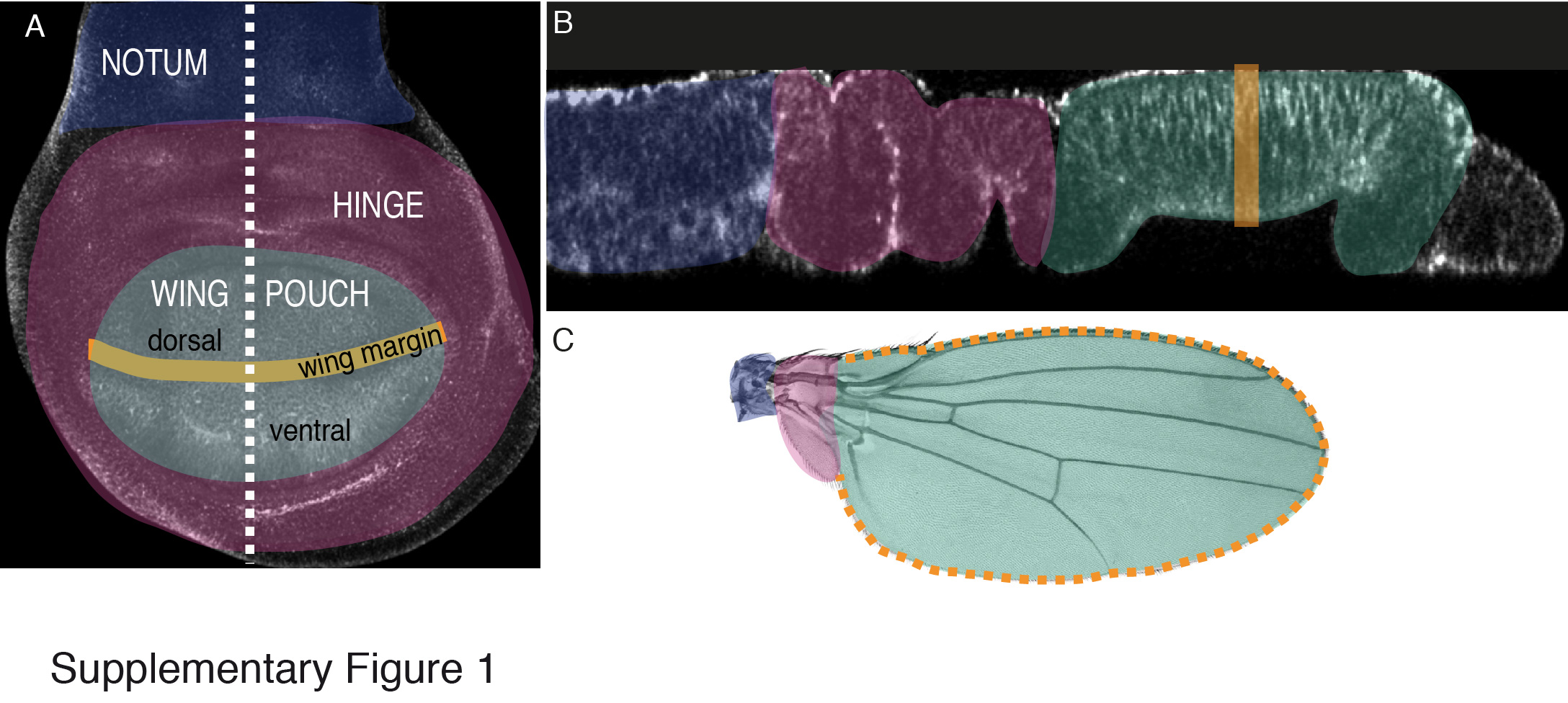

Supplement: Supplementary file 2 [file Image1.JPEG]

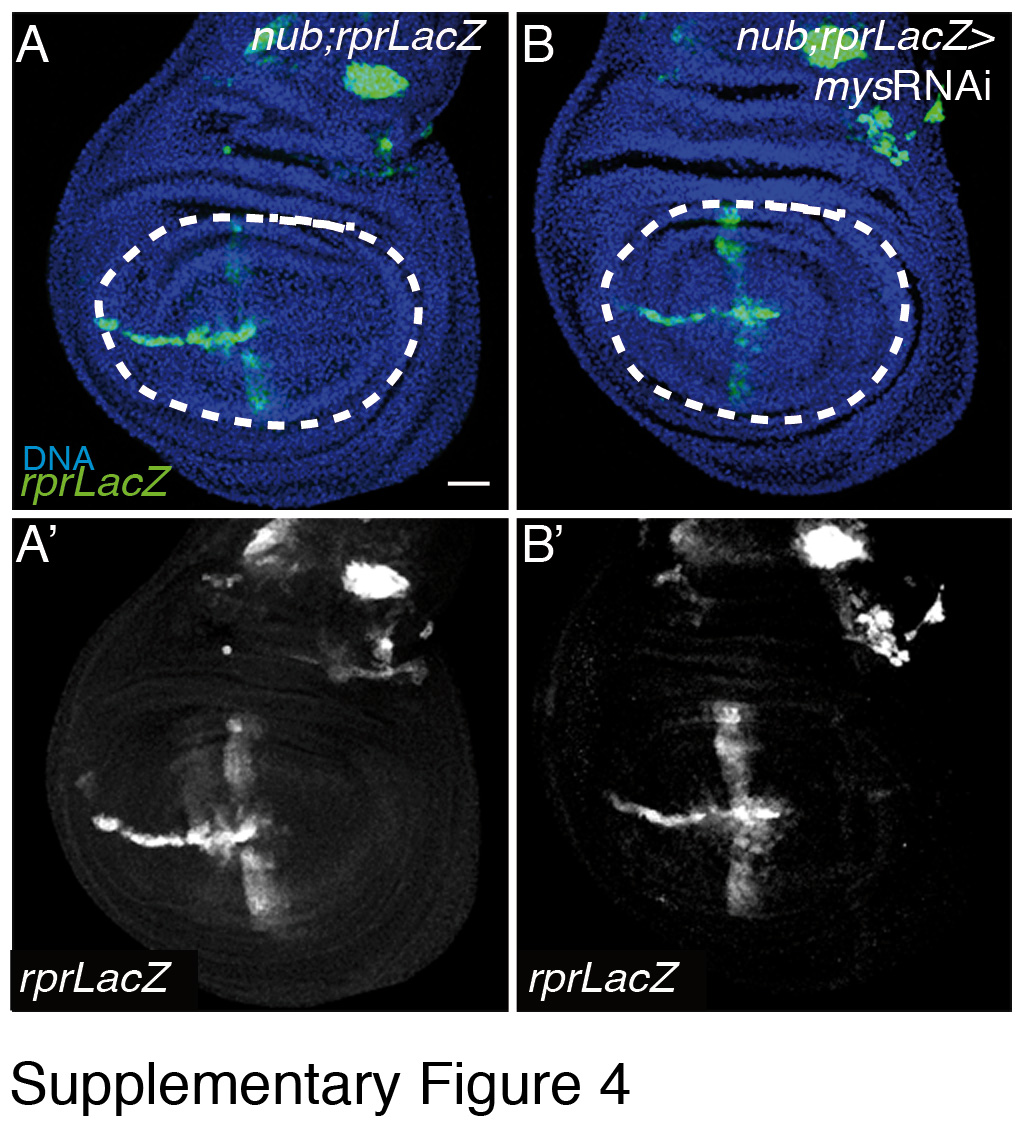

Supplement: Supplementary file 3 [file Image4.JPEG]

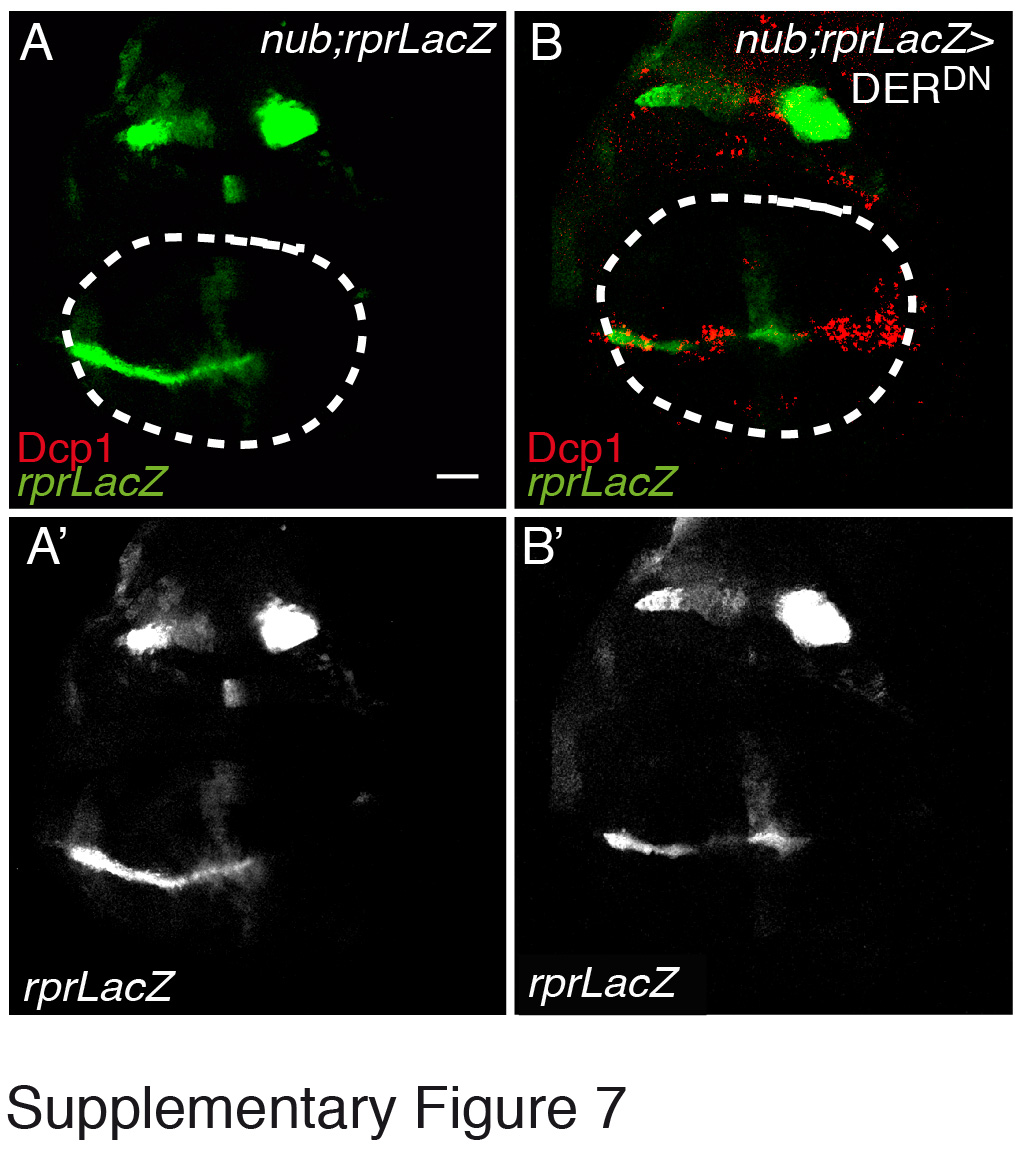

Supplement: Supplementary file 4 [file Image7.JPEG]

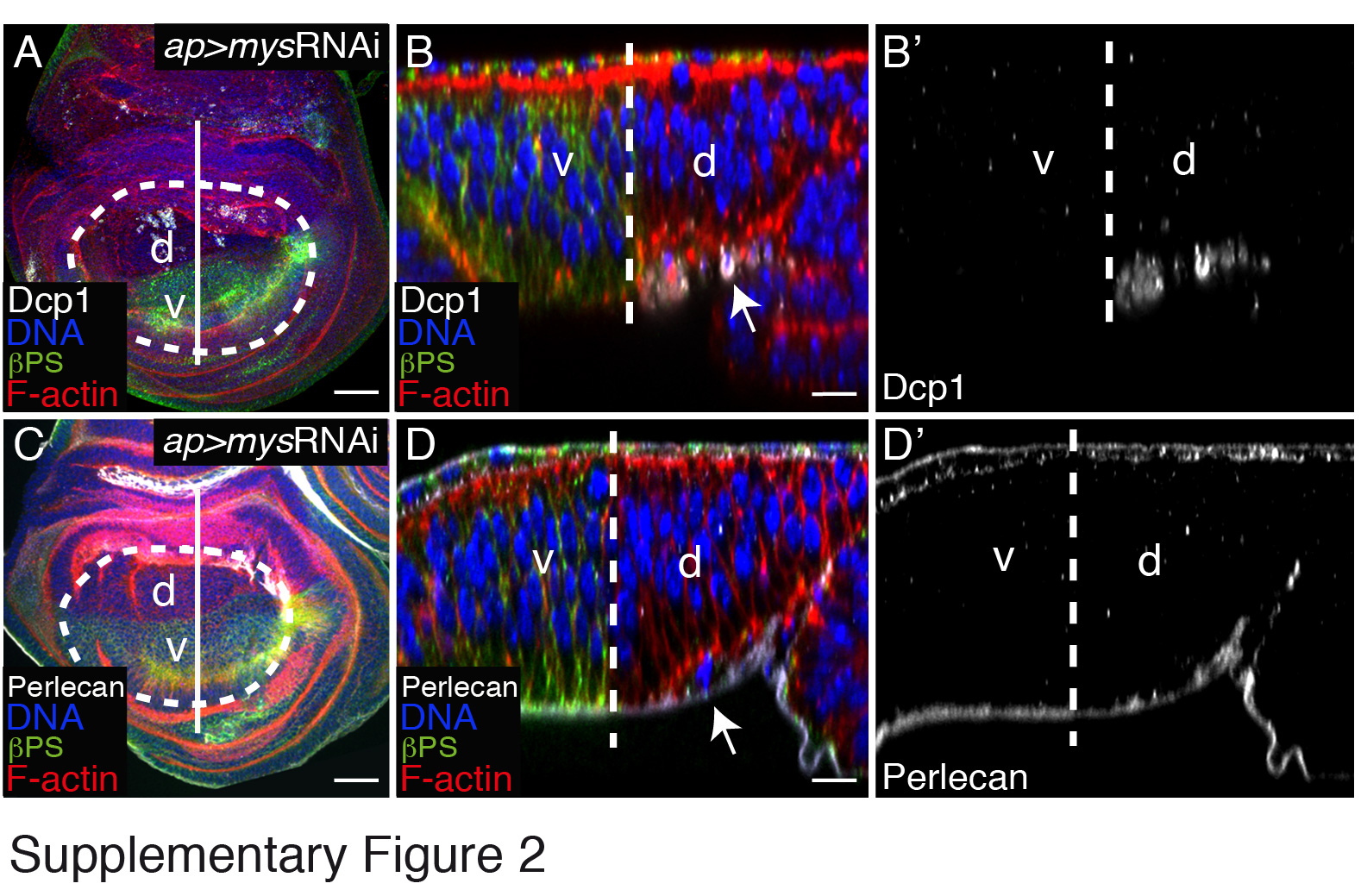

Supplement: Supplementary file 5 [file Image2.JPEG]

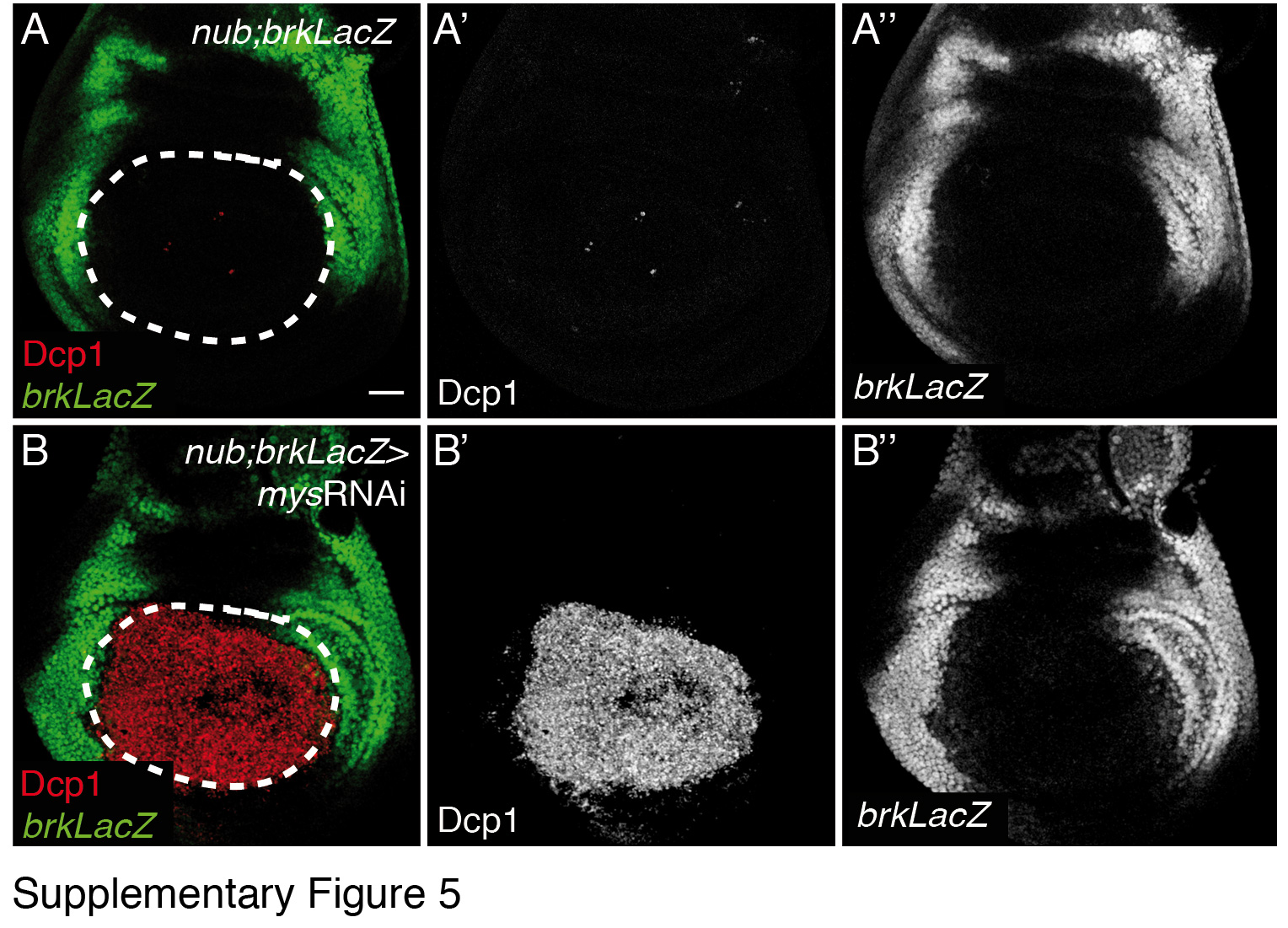

Supplement: Supplementary file 6 [file Image5.JPEG]

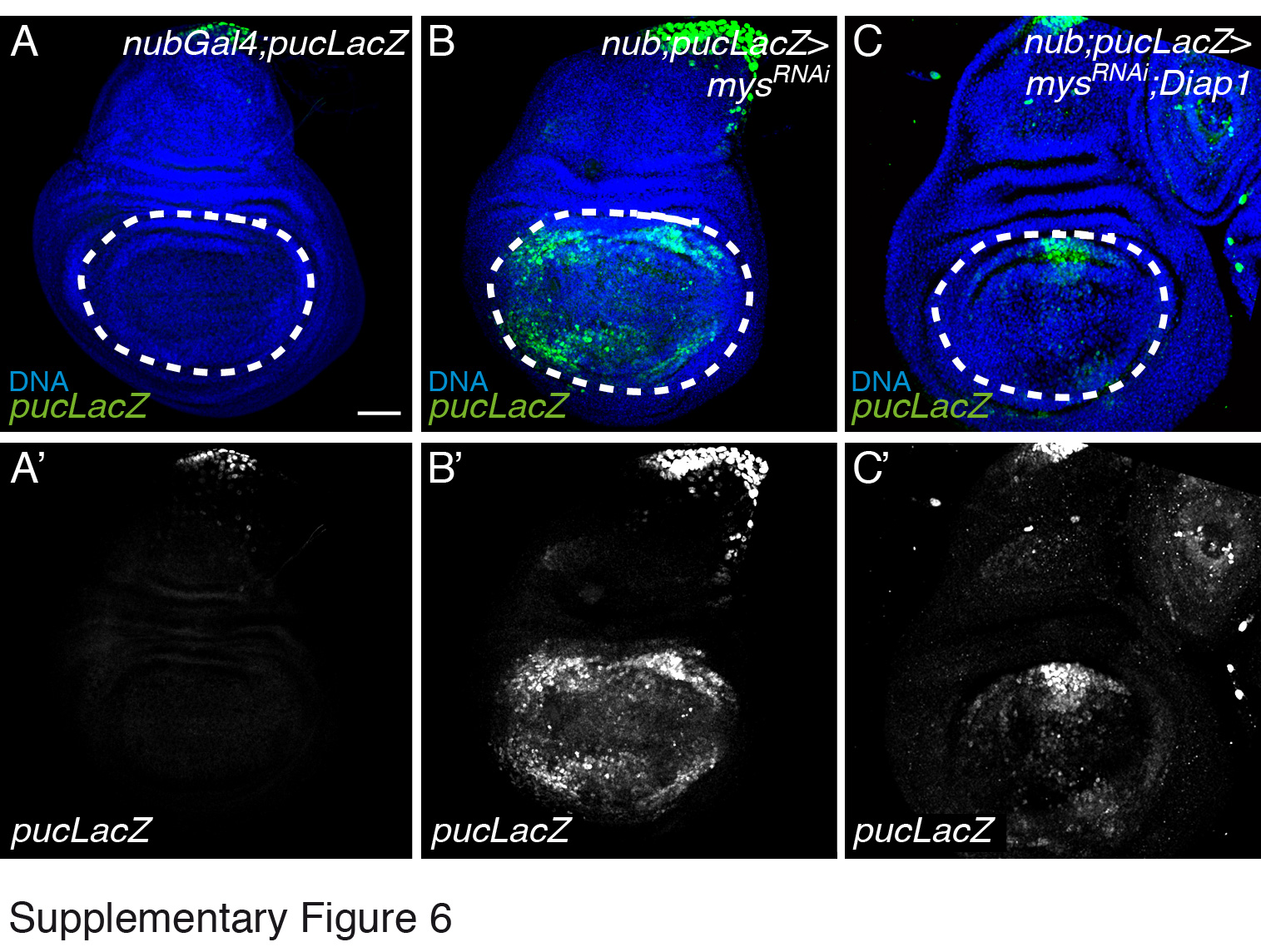

Supplement: Supplementary file 7 [file Image6.JPEG]
